# Increased risk of breast cancer among female relatives of patients with ataxia-telangiectasia: a causal relationship?

**DOI:** 10.1038/sj.bjc.6602786

**Published:** 2005-09-13

**Authors:** A K d'Almeida, E Cavaciuti, M-G Dondon, A Laugé, N Janin, D Stoppa-Lyonnet, N Andrieu

**Affiliations:** 1 Inserm Emi 00-06 & Service de Biostatistiques, Institut Curie, 26 rue d'Ulm, 75248 Paris Cedex 05, France; 2 Inserm IC10213 & Service de Biostatistiques, Institut Curie, 26 rue d'Ulm, 75248 Paris Cedex 05, France; 3 Service de Génétique Oncologique, Institut Curie, 26 rue d'Ulm, 75248 Paris Cedex 05, France; 4 Département de Génétique Humaine, CHU Sart Tilman, 4000 Liège, Belgium

**Sir**,

We read with much interest the paper by Olsen *et al* (2005), in which they observed an increased risk for early-onset breast cancer in a follow-up study of the incidence of cancer in 1445 blood relatives of 75 patients with Ataxia-Telangectasia diagnosed in Denmark, Norway, Finland and Sweden. The results of this study are supported by the unique study design in which AT patients were identified from medical records, and relatives were identified through population registry and validated for cancer, resulting in 60 years complete follow-up of the entire study population. The excess risk for breast cancer was evident only in the mothers of AT patients and they found no increase in breast cancer incidence by increasing the probability of being a mutation carrier. Their findings questioned the hypothesis of a causal relationship with ATM heterozygosity, which is the assumption of a number of past and ongoing studies (e.g. Bernstein *et al*, 2003).

Olsen *et al* mentioned that their findings were consistent with our study showing that the risk for breast cancer among female relatives seems to be restricted to the subgroup of obligate carriers (Geoffroy-Perez *et al*, 2001). They also mentioned that mutation carrier testing among families may bias the estimates by selective testing of survivors and/or relatives affected by cancer. In our French family study, we collected DNA samples from 401 individuals out of the 1423 relatives. This allowed us to classify 412 extra individuals as either carriers or noncarriers, allowing us to classify 70% of the breast cancer cases (20 out of 28) and 56% of the unaffected female relatives (300 out of 683). Therefore, we wondered about the potential bias of the relative risk estimates due to differential genotyping of cases compared to unaffected relatives. Indeed, the overgenotyping of cases may have biased the results towards the null hypothesis within the categories of relatives with uncertain genotype, resulting in a lack of gradient in breast cancer incidence in our study when using the ‘mixed approach’ (Geoffroy-Perez *et al*, 2001). Therefore, we reanalysed our data ignoring the genotyping (i.e. relatives were categorised according to their *a priori* probability of being a mutation carrier, i.e. the ‘*a priori* probabilities’ method) and using the correction for genotyping as proposed in Olsen *et al* (2005) (i.e. the ‘corrected mixed approach’). The main design feature of our study and the genotyping of the AT locus have been previously described (Janin *et al*, 1999). We estimated the standardised incidence ratio (SIR) of breast cancer as for Cavaciuti *et al* (2005). For this letter, we calculated the expected number of cancers per 5-year age category using the updated French age-, sex- and period-specific (1978–1982, 1983–1987, 1988–1992 and 1993–1997) estimated incidences (Remontet *et al*, 2003).

The results showed that, although more precise, genotyping (or the mixed approach) led to a point estimate of breast cancer risk among carriers lower than that calculated using either the *a priori* probabilities (SIR=4.48) or the corrected mixed approach (SIR=5.13) (Table 1). Moreover, when using the *a priori* probabilities, although none of the SIRs were significant, the excess risk for breast cancer did not seem to be restricted to the subgroup of carriers. Indeed, we found a gradient of breast cancer incidence with increasing probability of being a mutation carrier. We found an increased risk of breast cancer among relatives, with a 12.5% probability of being a carrier. This was mostly explained by an oversampling of the offspring of the AT patient's great-aunts or great-uncles when one of the offspring was diagnosed with cancer (Table 2). When we excluded these offspring, we found a *P*=0.012 for the trend. In the corrected mixed approach, there was no clear gradient of point estimate, even borderline, with a *P*=0.048 for the trend. The lack of gradient observed in the corrected mixed approach may be because of a residual bias due to the selective testing being insufficiently corrected by the method of Olsen *et al*. Overall, using the method proposed by Thompson and Easton (2001) to calculate the relative risk of breast cancer associated with being a carrier, weighted with the *a priori* probability of being a carrier, we found that the risk varied very little irrespective of the method used (Table 1).

Similar to what was seen by Olsen *et al*, the association with breast cancer in our study appeared particularly strong in the group of mothers compared to aunts or grandmothers, even after accounting for their 50% probability of being a carrier. We estimated an SIR of 7.1 (95% CI: 1.4–21) (Tables 1 and 2), which was similar to the SIR of 6.7 (95% CI: 2.9–13) found by Olsen *et al*. However, we cannot rule out an association in the group of carrier female relatives other than mothers. Indeed, the mixed approach gave a significantly increased risk of breast cancer of 3.2 (95% CI: 1.2–6.9) and the corrected mixed approach gave an increased, but not significant risk of 2.9 (95% CI: 0.04–16) (Table 1). None of the heterogeneity tests were significant. However, sample size of the group of carrier relatives other than mothers was very small for both approaches. Surprisingly, the recently published study on 1160 relatives of 169 UK AT patients did not observe a significant excess risk of breast cancer in mothers (SIR=1.87; 95% CI: 0.61–4.36). The highest excess risk observed in this study was in the aunts (Thompson *et al*, 2005). However, the percentage of mothers diagnosed with breast cancer in the UK study was particularly low (3.8% against 2.1% expected) compared to either the Nordic study (12.5% against 1.9% expected) or our study (8.1% against 1.1% expected), suggesting low participation of families with an ill or deceased mother.

Our findings are consistent with those of Olsen *et al* for a strong association with breast cancer in the group of mothers. When using an *a priori* probability approach, our findings were also consistent with the existence of a possibly weaker association in the group of carrier relatives other than mothers, and with the existence of a gradient in breast cancer risk with increasing probability of being a mutation carrier. Due to the small group sizes, it is not clear whether the association found in mothers was different from that found in carrier relatives other than mothers. Both retrospective and prospective international studies could help to determine whether or not mothers of AT patients have a higher risk of breast cancer than that conferred by being an AT heterozygote.

## Figures and Tables

**Table 1:** Breast cancer risk estimates according to mutation carrier probabilities

	**Mixed approach as in** **Geoffroy-Perez *et al* (2001)**						***A priori* carrier probabilities**						**Corrected mixed approach as proposed by** **Olsen *et al* (2005)**					
**Female relative**	**No.**	**PY**	**Obs**	**Exp**	**SIR**	**95% CI**	**No.**	**PY**	**Obs**	**Exp**	**SIR**	**95% CI**	**No.**	**PY**	**Obs**	**Exp**	**SIR**	**95% CI**
ALL	711	33 002.12	28	19.26	1.45	0.97–2.10												
																		
*Mutation carrier probability*																		
1	115	5075.3	9	2.32	3.88	1.77–7.36	41	1848.6	3	0.67	4.48	0.90–13.1	44	2030.3	4	0.78	5.13	1.38–13.1
0.5	198	8764.9	5	5.19	0.96	0.31–2.25	199	8895.8	8	4.44	1.80	0.78–3.55	318	13 823.0	9	7.52	1.20	0.55–2.27
0.25	108	5403.7	3	3.27	0.92	0.18–2.68	353	17 511.5	14	12.27	1.14	0.62–1.91	155	8365.1	10	5.52	1.81	0.87–3.33
0.125	5	194.4	0	0.06	—		102	3611.9	3	0.97	3.09	0.62–9.04	8	281.7	0	0.06	—	
0	285	13 563.8	11	8.42	1.31	0.65–2.34	16	1134.3	0	0.91	—		186	8502.1	5	5.38	0.93	0.30–2.17
			Weighted SIR		2.42	1.32–4.06					2.67	1.53–4.32					2.54	1.42–4.18
																		
Mother	37	1559.5	3	0.42	7.14	1.44–20.9												
Other carrier female relatives	78	3515.8	6	1.90	3.16	1.15–6.87	4	289.1	0	0.24	—		7	470.7	1	0.35	2.86	0.04–15.9

CI=confidence interval; SIR=standardised incidence ratio; PY=person-years.

**Table 2:** Breast cancer risk according to relationship to AT patient

**Relationship to AT patient**	**No.**	**PY**	**Obs**	**Exp**	**SIR**	**95% CI**
Mother	37	1559.5	3	0.42	7.14	1.44–20.9
						
*All relatives except mother*	670	31 167.4	25	18.53	1.35	0.87–1.99
Aunt	99	4033.6	2	1.26	1.59	0.18–5.73
Grandmother	65	4291.6	6	3.24	1.85	0.68–4.03
Grandaunt	158	9962.8	9	7.59	1.19	0.54–2.25
Great-grandmother	85	6374.2	5	5.34	0.94	0.30–2.19
Sister	28	475.9	0	0.03	—	
Cousin	116	2032.7	0	0.12	—	
Daughter of great-aunt or -uncle	81	2712.9	3	0.45	6.67	1.34–19.5
Other relationship^a^	38	1283.6	0	0.59	—	

a For example, great great grandmother, nephew.
